# Identifying Themes in the Knowledge and Attitudes of UK Healthcare Professionals Towards Transgender and Nonbinary Patients: An Evidence Review

**DOI:** 10.1111/tct.70367

**Published:** 2026-04-06

**Authors:** Amy Martin, Louise Preston

**Affiliations:** ^1^ Yorkshire and Humber School of Public Health Leeds UK; ^2^ University of Sheffield, School of Medicine and Population Health Sheffield UK

**Keywords:** health inequalities, healthcare access, healthcare inclusion, transgender

## Abstract

**Background:**

Trans (trans) is an umbrella term referring to diverse gender identities including transgender and nonbinary. Trans people have faced discrimination in healthcare settings as demonstrated in research reporting the experiences of trans patients. There is limited UK‐based literature on this topic, particularly from healthcare professional (HCP) perspectives. This review draws together existing evidence on themes in the attitudes and knowledge of HCPs towards trans patients.

**Methods:**

In this evidence review, a systematic search was conducted of multiple key databases using key concepts: (1) transgender, (2) attitudes and (3) healthcare professionals. Reflexive thematic analysis was used to analyse the qualitative data and narrative synthesis for the quantitative data. MMAT checklists were used to critically appraise the data.

**Findings:**

After thematic analysis of 14 articles, five themes were developed: (1) discriminatory attitudes towards trans patients, (2) knowledge gaps and training needs, (3) uncertainty around caring for trans patients, (4) impact of HCP knowledge and attitudes on trans patients, (5) trans inclusive attitudes of HCPs.

**Conclusion:**

The main themes were interlinked: Discriminatory attitudes (Theme 1) were connected to knowledge gaps (Theme 2), which were linked with uncertainty (Theme 3), ultimately all negatively impacting trans patients (Theme 4). Recommendations such as embedding trans healthcare education in HCP curriculums and implementing trans‐inclusive policy were made. Recommendations from these findings aim to contribute to a more trans‐inclusive healthcare culture where trans patients feel welcome instead of stigmatised and can build trusting HCP–patient relationships with positive consequences for patient care.

## 1 Background

Transgender (trans) is an umbrella term for individuals whose gender is not the same as the sex they were assigned at birth and can apply to diverse gender identities including but not limited to transgender, nonbinary and genderqueer [1, 2]. As per the latest Census, the first to ask about gender identity, there are an estimated 262,000 trans people in the United Kingdom. Thus, trans people represent 0.5% of the UK population [3]. Trans‐inclusive healthcare is a poorly understood, divisive and highly politicised topic in the United Kingdom. Trans people are often stigmatised, and there has been a measurable antitrans movement in recent years [4].

Trans patients in healthcare settings report mistrust, hostility and disdain from HCPs [5]. This has been linked to consequences for patients such as fear, avoidance and mistrust of HCPs and healthcare services [6, 7, 8, 9]. For instance, an investigation by TransActual [10], the national UK organisation working to improve trans people's experience of healthcare, found that 70% of trans people experienced transphobia when accessing healthcare and 57% of trans people avoid going to the GP when they are unwell [10]. A National LGBT (lesbian, gay, bisexual, trans) survey was conducted in the United Kingdom of 108,100 people and 13% were trans. Of trans respondents in healthcare settings, 21% reported that their needs were ignored, 40% reported negative experiences, 18% reported experiences of inappropriate curiosity from HCPs, and 18% avoided treatment because they feared discrimination [11]. Intersectionality is a vital aspect of trans patients' experiences of healthcare [12] as those with intersecting marginalised identities are vulnerable to the compounded impacts of different forms of discrimination. For example, 53% of trans people of colour and 60% of trans disabled people experienced racism and ableism respectively in healthcare settings [10].

Trans‐inclusive healthcare education is not widely embedded in the curriculum for HCP undergraduates or postgraduates [13, 14, 15, 16]. Some healthcare training bodies do not have trans‐inclusive healthcare in the curriculum at all [17], and governing bodies such as the General Medical Council (GMC) published specific guidance for the first time in 2021 [18].

There has historically been limited literature on the attitudes and knowledge of HCPs towards trans patients. This is likely due to longstanding discrimination towards trans people, which likely contributed to disincentivising historical research efforts. More recently, there has been a move away from pathologising trans patients in healthcare settings [5, 19] such as changes to diagnostic criteria and legal protections over ‘gender reassignment’ under the Equality Act 2010 [11].

No evidence reviews on this topic are currently published that are specific to a UK setting representing a gap in the evidence base. Understanding issues specific to the United Kingdom is important because there could be discrepancies between best practice guidance and real‐world beliefs and behaviours. The factors influencing trans‐inclusive healthcare are complex and multifactorial including healthcare policy, legislation, political will and funding, societal attitudes and other systemic and institutional manifestations of transphobia [10, 20]. However, HCP attitudes and knowledge are key drivers influencing trans patient experiences. The aim was to review existing evidence to better understand the attitudes and knowledge of HCPs towards transgender patients in the United Kingdom.

## 2 Methods

### 2.1 Context

This evidence review was completed as a Masters in Public Health dissertation, and as such, AM carried out the methods independently under the supervision of LP.

### 2.2 Identification of Relevant Studies

A systematic search of relevant databases was conducted (MEDLINE, CINAHL, PsycINFO, ERIC and Web of Science). Strategies were used to enhance the sensitivity of the search such as truncation and Boolean operators. Synonyms for three key concepts—(1) ‘transgender’, (2) ‘attitudes’ and (3) ‘healthcare professionals’ were developed using resources from reputable organisations such as Stonewall and the NHS. Terminology has evolved in recent decades [21, 22, 23], and efforts were made to incorporate this. A full list of the search terms and example search strategy is in Supporting Information.

Articles for inclusion were limited to UK settings at the screening stage to allow synthesis of evidence from comparable healthcare systems, infrastructure, training and culture, which likely impact knowledge, attitudes and healthcare practice. No date limits were applied to avoid restricting an already small pool of research. Ancestry searching and citation searching were used alongside grey literature searching to identify any reports or unpublished evidence related to the topic.

### 2.3 Screening and Selection

Search results were screened by AM and subject to a sequential screening process using the predetermined eligibility criteria (see Table 1). Title and abstract screen was followed by full text screen, where indicated, if fulfilment of screening criteria was ambiguous. References were extracted and deduplicated using Zotero reference management software.

**TABLE 1:** Inclusion and exclusion criteria.

	Inclusion criteria	Exclusion criteria
Setting	Studies based in any healthcare setting in the United Kingdom.	Studies not based in a healthcare setting. Studies outside the United Kingdom.
Perspective	Any type of healthcare professional was included such as doctor, nurse, midwife and physiotherapist.	Healthcare students, non‐healthcare staff in a healthcare setting (e.g., estates and facilities and IT support).
Interest (phenomenon of)	Studies with a focus on trans and nonbinary patients inclusive of the diversity of gender identities.	Studies focused on trans and nonbinary patient experiences not related to healthcare interactions. Studies focused on experiences of sexuality rather than gender identity.
Evaluation	Studies on the attitudes and knowledge of HCPs. Any type of study.	Studies from a patient perspective.

### 2.4 Data Extraction, Synthesis and Reporting

A data extraction form adapted from a BMJ Open resource [24] was used for each article in the review. This evidence review was completed as a postgraduate dissertation by AM, independently, in line with the course requirements. However, in preparing the article for publication, data checking of 10% of included studies was undertaken by LP. No discrepancies were identified.

The Mixed Methods Appraisal Tool (MMAT) [25] was used, as it is designed for appraisal in mixed methods evidence reviews. The organisational report was critically appraised using the AACODS (Authority, Accuracy, Coverage, Objectivity, Date, Significance) checklist, which was designed for grey literature [26].

Qualitative data were synthesised using reflexive thematic analysis [27, 28]. This allowed an in‐depth and iterative analysis process of code generation and collation, and development, refinement and finalising themes. The reflexive aspect builds on the original framework by Braun and Clarke, going further to recognise the inherent influence that a researcher has on theme development, as an expectation rather than a drawback. A codebook was kept with Microsoft Word and Excel. Quantitative data were integrated using narrative synthesis. Supervision meetings enhanced trustworthiness via opportunities for reflexivity and to engage in critical thinking.

### 2.5 Reflexivity Statement

Authors take a gender affirming stance and have considered how this has influenced interpretation and presentation of these findings in line with this. An intersectional lens is vital to the authors; however, the inconsistent availability of demographic information or analysis in the articles limited conclusions that could be drawn in this respect. Regular supervision encouraged opportunities for reflexive discussion.

## 3 Results

### 3.1 Study Selection

**FIGURE 1:**
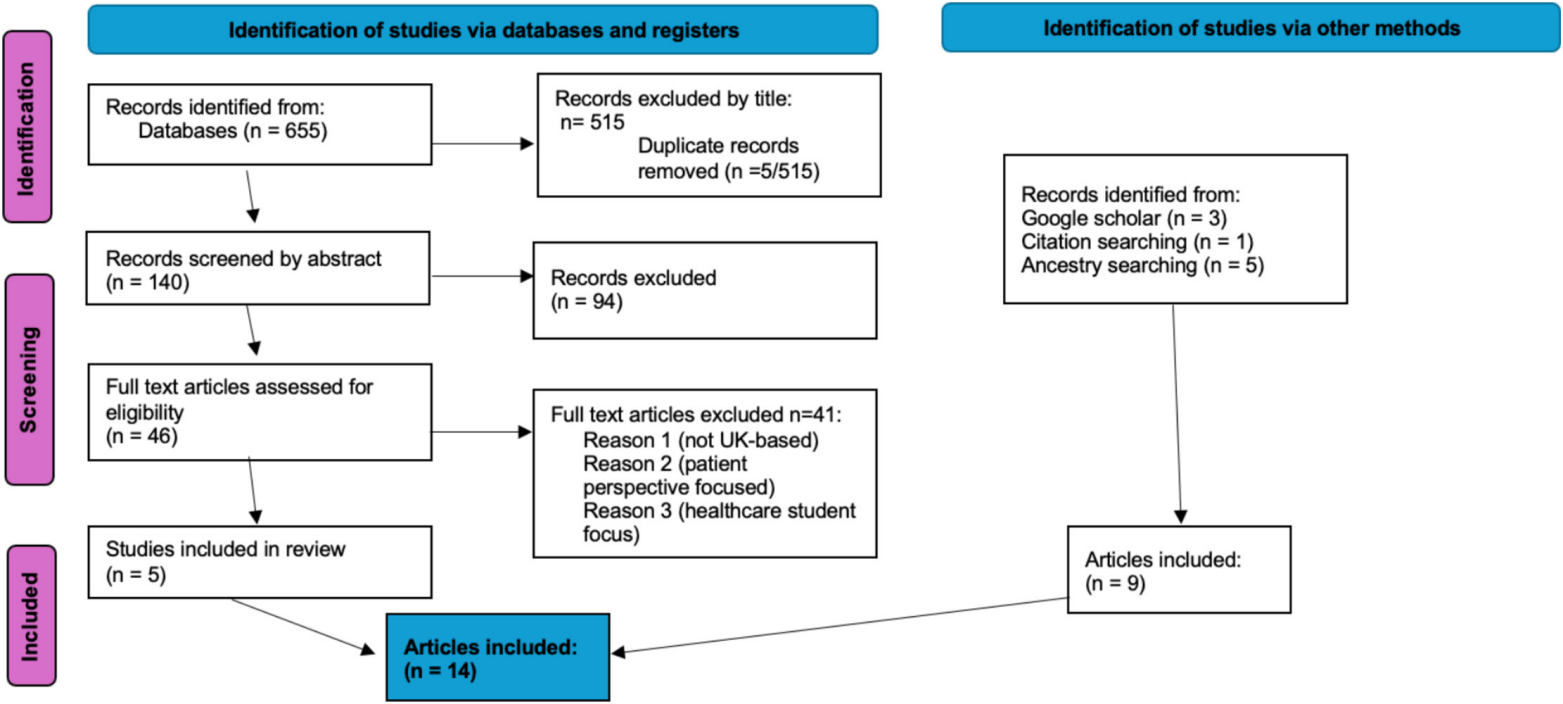
PRISMA diagram.

The PRISMA diagram in Figure 1 outlines the results (*n* = 14) of the screening process.

### 3.2 Study Characteristics

#### 3.2.1 Overall

Despite no date limit, the selected articles were published between 2014 and 2023 with most (10/14) published from 2020 onwards. Eight were qualitative, two quantitative and four mixed methods design. The sample sizes ranged from 5 to 3001, though most (9/14) were smaller studies with sample sizes under 30.

#### 3.2.2 Setting

All studies were based in the United Kingdom, half in England. The healthcare settings varied and included Oncology, Sexual Health, Mental Health, Maternity and Primary Care.

#### 3.2.3 Perspective

Many different HCPs were included, such as doctors, nurses, counsellors, health care assistants, mental health care practitioners and sexual health practitioners.

#### 3.2.4 Interest

The majority of articles (9/14) were trans‐specific, and the remaining (5/14) had a broader LGBTQ+ focus with some disaggregated data.

The same participants were used in Canvin [29] and Canvin et al. [30]. More data are provided in Canvin [29], the PhD thesis of the author, offering additional insight. Similarly, findings from Mikulak [31] and Mikulak et al. [32] were based on mostly the same participants. However, the sample in Mikulak et al. [32] used data from an additional two participants for reasons unspecified. See Table 2 for a summary of study characteristics.

**TABLE 2:** Study characteristics.

	Study reference	Study design	Setting	Population/perspective	Sampling methods	Interest	Evaluation	Data collection	Data analysis
Bashir, S., Fend, M. and Sarfraz, M.A., 2023.	[33]	Quantitative (descriptive)	SE^a^ England Doctors in physical and mental health settings	72 doctors (trainees and consultants)	Purposive sampling	Trans patients	Clinicians' views	Online survey	Summary descriptive statistics
Berner, A.M., Hughes, D.J., Tharmalingam, H., Baker, T., Heyworth, B., Banerjee, S. and Saunders, D., 2020.	[34]	Quantitative survey (descriptive)	UK Oncology (secondary care)	258 UK oncology trainees and consultants	Purposive and volunteer sampling (email and social media advertising)	LGBTQ+^b^ patients (some disaggregated trans‐specific findings)	Attitudes and behaviours of oncologists	Online survey to assess self‐perceived knowledge	Descriptive statistics. Multifactorial ordinal logistic regression was utilised for Likert scale responses and Fisher's exact test for categorical responses. Holm‐Bonferroni multiple testing correction was applied, and all *p* values adjusted.
Braybrook, D., Bristowe, K., Timmins, L., Roach, A., Day, E., Clift, P., Rose, R., Marshall, S., Johnson, K., Sleeman, K.E. and Harding, R., 2023.	[35]	Qualitative	England	27 HCPs (doctors, nurses, social workers) (*n* = 74 total—mixed sample of clinicians, patients, and family members)	Purposive sampling	LGBTQ+ patients (some disaggregated trans‐specific findings)	Clinicians' views	Semistructured interviews	Reflexive thematic analysis
Brown, M., McCann, E., McLoughlin, G., Martin, C.H. and McCormick, F., 2023.	[36]	Mixed methods	UK Midwifery	29 Midwives	Purposive sampling	LGBT+ patients (4 trans‐specific quotes)	HCP views	Semistructured interviews Online survey	Thematic analysis Summary statistics
Canvin, L., 2020.	[29]	Qualitative	England Mental health settings	7 mental health professionals Age 27–38 5 female, 2 male 4 white British, 1 white, 1 British, mixed Caucasian and East Asian All cisgender 5 straight, 1 demisexual, 1 undisclosed	Purposive sampling	Gender diverse patients	HCP views of working with gender diverse adults	Semistructured narrative interview Face to face, telephone, online	Constructionist narrative analysis
Canvin, L., Twist, J. and Solomons, W., 2022.	[30]	Qualitative	England Mental health settings	7 HCPs (2 clinical psychologists, 1 trainee clinical psychologist, 1 consultant psychiatrist, 1 mental health nurse, 1 social worker, 1 art therapist)	Purposive Social media recruitment	Gender diverse patients	HCP views of working with gender diverse adults	Semistructured narrative interview Face to face, telephone, online	Constructionist narrative analysis
Kirlew, M.I., Lord, H. and Weber, J., 2020.	[37]	Qualitative	England Health and social care professionals	5 HCPs (disciplines unspecified)	Volunteer	Trans patients	HCP perspectives on caring for trans patients	Semistructured interviews	Thematic analysis
Lefkowitz, A.R. and Mannell, J., 2017.	[38]	Qualitative	England	20 Sexual health service providers	Purposive sampling of 86 UK sexual health clinics	Trans patients	Perceptions of service providers towards trans patients (youth)	Semistructured interviews	Thematic analysis
Mikulak M. 2021	[31]	Qualitative	UK Primary care	18 health professionals (7 GP^c^, 5 mental health professionals, 3 GIC^d^ specialists, 1 voice therapist, 1 practice nurse, 1 oncologist)	Purposive and snowball	Trans patients	Examples of HCP ignorance and influence on practice	Semistructured telephone interviews	Thematic analysis And modified grounded theory
Mikulak, M., Ryan, S., Ma, R., Martin, S., Stewart, J., Davidson, S. and Stepney, M., 2021	[32]	Qualitative	UK Primary care	20 health professionals (7 GP, 1 oncologist, 1 practice nurse, 2 counsellors, 2 psychologist, 3 mental health practitioner, 2 psychiatrist, 1 registrar, 1 voice therapist) 16 cis, 3 trans, 1 nonbinary	Purposive and snowball	Trans patients	HCP perspectives on barriers to trans healthcare	Semistructured telephone interviews	Thematic analysis And modified grounded theory
Mollitt, P.C., 2022b.	[39]	Mixed methods	UK Counsellors	576 counsellors	Purposive sampling	Trans patients	HCP attitudes	Online survey	Reflexive Thematic analysis
Owen‐Pugh, V. and Baines, L., 2014.	[40]	Qualitative	England	16 Counsellors	Purposive, and snowball sampling. Inclusion criteria: ‘novice counsellors’ (up to 5 years from qualification)	LGBTQ+ patients (some disaggregated trans‐specific findings)	Experiences of counsellors with LGBTQ+ patients	Semistructured interviews (face to face and email interviews)	Grounded theory
Simpson, P., Almack, K. and Walthery, P., 2018.	[41]	Mixed methods	England Care homes	*N* = 187 Unclear how many HCPs vs. managers	Purposive sampling	LGBT patients (2 trans‐specific findings only)	Care home staff views	Online and in‐person survey	Unclear
Somerville, C., 2015.	[42]	Mixed methods	UK Healthcare settings	3001 health and social care staff	Volunteer sampling	LGBT (with disaggregated findings specific to trans patients)	HCP knowledge, attitudes, and behaviours	Online survey	Narrative summary Descriptive statistics

^a^ South East.

^b^ Lesbian, gay, bisexual, trans, queer+.

^c^ General practitioner.

^d^ Gender Identity Clinic.

### 3.3 Quality Assessment

See Table 3 for a summary of the quality assessment of the empirical studies using the MMAT critical appraisal tool [25]. Most of the studies are of good methodological quality with 10/13 scoring 80% or above, two scoring 60% and one scoring 40%. Only two relevant quotes were extracted from the article that scored 40% [28], meaning it was not a key component of the review findings. The organisational report was critically appraised using the AACODS checklist and scored 6/6.

**TABLE 3:** MMAT critical appraisal summary.

	Article	MMAT^a^ score	Criteria met?				
			1	2	3	4	5
Quantitative	Berner et al. [34]	80%	Yes	Yes	Yes	No	Yes
	Bashir et al. [33]	60%	Yes	No	Yes	No	Yes
Qualitative	Lefkowitz [38]	100%	Yes	Yes	Yes	Yes	Yes
	Mikulak et al. [32]	100%	Yes	Yes	Yes	Yes	Yes
	Mikulak [31]	100%	Yes	Yes	Yes	Yes	Yes
	Canvin [29]	100%	Yes	Yes	Yes	Yes	Yes
	Canvin et al. [30]	100%	Yes	Yes	Yes	Yes	Yes
	Braybrook [35]	100%	Yes	Yes	Yes	Yes	Yes
	Owen‐Pugh and Baines [40]	80%	Yes	No	Yes	Yes	Yes
	Kirlew et al. [37]	80%	Yes	Yes	No	Yes	Yes
Mixed methods	Mollitt [39]	100%	Yes	Yes	Yes	Yes	Yes
	Simpson et al. [41]	60%	No	Yes	Yes	No	Yes
	Brown et al. [36]	40%	No	Yes	No	No	Yes

^a^ Mixed Methods Appraisal Tool.

## 4 Themes

Five main themes resulted from reflexive thematic analysis of the qualitative studies. Relevant quantitative evidence is integrated in the text.

1. Discriminatory attitudes towards trans patients
2. Knowledge gaps and training needs of healthcare professionals
3. Uncertainty around caring for trans patients
4. Impact of healthcare professionals' attitudes and knowledge on trans patients
5. Trans inclusive attitudes of healthcare professionals

### 4.1 Theme 1: Discriminatory Attitudes Towards Trans Patients

Stigmatisation and pathologisation of trans identities by HCPs was present in most of the studies [29, 30, 31, 32, 33, 36, 37, 38, 42, 43]. HCPs across various settings reported beliefs that trans identities are ‘not a real thing’ [32, 33] and 22.5% of doctors surveyed (*n* = 72) [33] agreed with the statement ‘transgender men are only able to look like but not be men’. Further, only two sexual health practitioners (*n* = 20) felt that young people should be believed when they say that they are trans, reflecting a prevailing attitude of minimisation of trans identities [38].

Moreover, trans identities were conflated with mental illness [30, 37, 38, 42, 43] or described as a cause of mental illness [29, 36, 38] in several studies. Even in mental health settings, 6% of counsellors that had worked with trans patients (*n* = 520) described gender diversity as psychological illness, including personality disorder, and a further 12% were unsure [43]. One sexual health practitioner was bolstered by historical psychiatry guidance: ‘… the DSM diagnostic manual … regards trans … as a potential mental health issue … I would agree with that’ [38].

In the largest survey in this review, 20% of HCPs with varied roles in patient care (*n* = 1800) had witnessed colleagues make negative comments towards trans patients, including intentional misgendering [42]. Discrimination was a main theme identified by Kirlew, Lord and Weber [37], and most HCPs (*n* = 5) had witnessed discrimination towards trans patients such as *derogatory impersonations* [37]. Across the studies in almost all instances of acknowledged discrimination, HCPs described themselves as observers rather than perpetrators. HCPs observed discriminatory attitudes around ward preferences in secondary care settings [30, 37, 42], name changes [32] and referrals [31]: ‘… [some GPs] ask lots of really inappropriate questions and hold back referrals’ [31]. HCPs in several studies described observing paternalistic attitudes, including making decisions for trans patients without consulting them, particularly around toilet preference and ward placement [29, 31, 37, 38, 42].

### 4.2 Theme 2: Knowledge Gaps and Training Needs

It was common across the studies that HCPs expressed or demonstrated low knowledge around trans‐inclusive healthcare and gender diversity, particularly nonbinary [32], and mainly used binary terms such as *sex change* [29]. For example, 18 Sexual Health practitioners (*n* = 20) used descriptions such as ‘male to female’ [38]. Furthermore, 87% of oncologists (*n* = 258) [34] always or often assumed that a patient is cisgender and 59% never ask a patient's gender identity (ibid). HCPs also confused gender and sexuality with only 15% of Sexual Health practitioners (*n* = 20) [38] distinguishing the two: ‘… transgender is when you're questioning your sexuality …’ [38].

Appropriate pronoun use was highlighted as *scary, awkward* [35] and challenging by HCPs in half of the studies [29, 32, 33, 34, 36, 37, 42]. Of oncologists surveyed (*n* = 258), 64% never ask a patient's pronouns [34], akin to 77.5% of doctors surveyed (*n* = 72) [33]. Primary care practitioners reported difficulties using ‘they’ in the singular [32] citing grammatical issues as it *‘*goes against your English education*’*. HCPs were also worried about offending patients [35, 37], yet only 22% of doctors (*n* = 72) were familiar with NHS policy on how to refer to trans patients in clinical records [33].

Knowledge gaps also extended to healthcare needs of trans patients, demonstrated by 24% of oncologists (*n* = 258) who incorrectly believed that trans and cis patients do not have different cancer risk factors [34], and 49% were unsure how to refer a trans patient for fertility treatment (ibid). Consultants were significantly more likely (*p* = 0.000157) to know this than trainee doctors (53% vs. 30%) [34]. Most HCPs felt that their training had left them unprepared to care for trans patients. Only 11% of HCPs (*n* = 2500) reported receiving any training during their healthcare careers about trans‐inclusive healthcare [42]. The content of the training sessions was inconsistent, but the most common topic was antidiscrimination policies. One of the least common topics (experienced by 19% of HCPs) was trans‐inclusive healthcare practices such as appropriate language [42]. Further, 44% of a sample of counsellors (*n* = 576) felt ill‐equipped to care for trans patients [43]. HCPs from medical oncology, primary care, counselling and nursing [32] identified their undergraduate and postgraduate training on trans‐inclusive healthcare as absent and *woefully inadequate* (ibid). One HCP described concerns that trans patients must ‘fill in [HCP knowledge] gaps that should already be filled’ [31].

### 4.3 Theme 3: Uncertainty Around Caring for Trans Patients

In a survey of HCPs across different disciplines and UK locations, only 24% of HCPs with direct patient caring roles (*n* = 1800) reported confidence in caring for trans patients. This finding differed by professional role—doctors were almost twice as likely as healthcare assistants to report that they are not confident meeting trans patients' needs [42]. Only 14% of oncologists surveyed were confident counselling trans patients on fertility issues [34]. One counsellor said that they ‘can't keep up’, which makes them feel *incompetent* [43]. Older HCPs were more likely to express uncertainty around trans‐inclusive healthcare than younger HCPs (ibid). This was contrasted by findings in Bashir, Fend and Sarfraz [33] who found higher levels of comfort communicating with trans patients in consultant doctors than junior doctors (ibid).

Several HCPs [30, 39] worried about saying the *wrong thing* [30], offending patients [35, 37, 43] or appearing ignorant [30]. Counsellors described reticence around treating trans patients because of the highly politicised climate in the United Kingdom and fear of litigation: ‘I could face professional ostracization or malicious complaints’ [43]. GPs were also reluctant to treat trans patients because of the *risks* [31]. Uncertainty was often paired with reluctance to treat trans patients and disengagement with professional development on trans healthcare.

### 4.4 Theme 4: Impact of Healthcare Professionals' Knowledge and Attitudes on Trans Patients

HCP views on the impact on quality of care were described explicitly and implicitly. In one article, 79% of oncologists (*n* = 258) thought that the HCP–patient relationship was impacted by communication around gender identity [34], and sexual health practitioners avoided asking trans patients questions relevant to their care, potentially leading to incorrect procedures [38]. HCPs described feeling *uncomfortable touching* trans patients [37], particularly amid gender‐affirming treatment [38], which another HCP recognised as an absence of ‘very basic care’ [37]. Moreover, concerns around safety were prominent in HCPs in secondary care who described isolating trans patients for *safety* reasons [29]. Several HCPs described trans patients as *unsafe* on hospital wards [29, 30, 35, 36], particularly male wards [29, 30].

Of oncologists surveyed, 57% felt that access to healthcare was impacted by gender identity [34]. Doctors described the NHS *blind spot* [32, 35] around cancer screening, which risks excluding trans patients who change their gender identity on their NHS records. HCPs avoid treating trans patients [30, 31, 32, 43] and refer inappropriately to Gender Identity Clinics (GICs) [29] or mental health services because they ‘don't know what to do’ [30]. Some HCPs in primary care obstructed referrals to GICs, obstructing timely access to care, because they felt it was their choice to first decide whether patients are trans [31]. One therapist [43] *removed* themself from the trans *debate*, framing this as a choice whether to treat trans patients. The implications of this ‘choice’ were highlighted by one HCP [31] who described GPs refusing to prescribe gender affirming care as a ‘dire situation, that puts people at risk’. HCPs in two studies observed trans patients avoiding clinical care interactions and appointments because of fears of discrimination from staff and patients [36, 37].

### 4.5 Theme 5: Trans Inclusive Attitudes of Healthcare Professionals

Although less common, trans inclusive attitudes were demonstrated: HCPs said they ‘treat everyone the same with dignity’ [37], *‘*accept differences without pathologising’ [39], and aimed to ‘push for more equality’ [29].

In a respectful way, ask what ward and toilets the trans patient would prefer. Is it really that difficult … ? [37]

HCPs who were happy to be described as an ‘LGBTQ+ friendly’ provider were more likely to be knowledgeable around trans‐inclusive healthcare (*p* = 0.039) [34]. Furthermore, 78% of counsellors felt that their stance towards trans patients was gender affirming [39]. HCPs described efforts to use preferred pronouns [36, 37], apologising [33] or correcting themselves when they mis‐stepped [30]. Several HCPs disagreed with colleagues who held discriminatory views [29, 30, 31, 32, 36, 37, 42] though, very few [29] described open disagreement or subsequent action. However, HCPs across several studies [37, 40], wanted to be *more informed* [43] about trans healthcare and to not burden trans patients with the responsibility to educate [36]. Some HCPs took accountability for their professional development by requesting national guidance [32], trying to *upskill*, seeking specialist [30] or community advice [31] and changing healthcare curriculums [36].

#### 4.5.1 Stratification of Results

Regarding temporal patterns, the four older articles published pre‐2020 were more likely to have a broader LGBT+ focus with limited trans‐specific disaggregated data. This perhaps reflects an increasing interest over time in differentiating research on sexuality and gender identity rather than a homogenous focus under the umbrella of ‘LGBT’. Furthermore, some of the most explicit transphobic comments were present in the articles published prior to 2020. This might reflect changes towards more inclusive attitudes but could also indicate a reticence to offer views openly due to perceptions of the professional risk of engaging in the increasingly ‘politicised’ discourse of recent years. In these aspects, there is not a clear picture from the available evidence and caution should be applied to these observations given the small number of articles included in this review. Four articles [35, 36, 40, 41] had comparatively limited relevant data. Two of these [36, 41] were also of poorer methodological quality than the other articles that were more significant in the synthesis.

## 5 Discussion

This review identified five themes characterising mostly negative attitudes of HCPs towards trans patients including discrimination, pathologisation and minimisation of trans identities. These negative attitudes were present across several healthcare settings including primary [31, 32] and secondary care [33, 42], as well as maternity [36], sexual health [38] and mental health services [43]. Discrimination was explicit, including derogatory ‘jokes’ about trans patients [37], and implicit, including a lack of trans‐inclusionary systems or policies. This was exemplified by issues around ward allocation for trans patients in secondary care [30, 37, 42] despite clear NHS policy, in place since 2019, instructing HCPs to accommodate trans patients' preferences [44] (as per the Equality Act 2010) [45]. This is mirrored in guidance from the British Medical Association [46]. However, because this review was conducted, this guidance may now be subject to change following UK Supreme Court ruling and the Equality and Human Rights Commission guidance [47].

The discrimination described at an individual level is at odds with best practice guidance for HCPs. The Nursing and Midwifery Council [48] states that HCPs must ‘avoid making assumptions’ and ‘recognise diversity and individual choice’ [48]. The high standards HCPs are held to are vital for equal access to healthcare where all patients are treated with respect and dignity [48]. Furthermore, multiple HCPs promoted treating trans patients ‘the same as’ anyone else [37, 41]. However, viewed through an equity rather than equality lens, it is most appropriate to give each patient what they need, not the same [49]. Given the additional barriers faced by trans patients, they would benefit from additional reassurance from HCPs that they are welcome.

The validity of the findings around uncertainty towards caring for trans patients, a key theme [29, 30, 31, 33, 42, 43], is strengthened by the alignment with literature from trans patients' perspectives that HCP's demonstrate uncertainty [49] and low knowledge [50]. Limited knowledge about diverse gender identities was a theme in every article (except Simpson et al. [41], where data were limited to two sentences). Most HCPs felt that they received inadequate or no education on trans health [31, 32, 37, 42]. Authors [40] described the poor knowledge as a ‘matter of concern’ owing to ethical issues of HCPs qualifying with insufficient knowledge to treat trans patients. There was a desire to be more informed amongst multiple HCPs [30, 34, 36, 37, 43], and almost all studies in this review called for improved education and training for HCPs on trans healthcare. A ‘knowledge lag’ is possible, where understanding on trans healthcare is changing faster than educational resources are updating. Some of the legal protections and terminology related to gender identity were not introduced widely in the United Kingdom until recent years [51]. Yet, continuing professional development is a cornerstone of health professions to maintain high standards of patient care. It is important that trans healthcare is a part of this given the ongoing changes and development in understanding and culture [52]. The landscape has changed significantly and some of the legal protections and terminology related to gender identity were not introduced widely in the United Kingdom until recent years [51]. This emphasises the importance of high‐quality trans inclusive education for HCPs at all levels of training including undergraduate and postgraduate.

### 5.1 Implications for Policy and Practice

Undergraduate and postgraduate training in the United Kingdom around trans‐inclusive healthcare is inconsistent, incomplete [35, 51] or absent [44]. Consistent high‐quality education is needed to improve the attitudes and knowledge of HCPs. Implementation of educational resources has been successful [53, 54] in reducing HCP discomfort [53] and increasing knowledge [55]. There have been successful examples of trial educational programmes with positive outcomes [53, 54] and of implementation of enhanced educational resources [55]. For example, when educational resources about gender identity and gender affirming care were added to healthcare curriculums, there were decreases in discomfort towards trans patients [53] and increases in knowledge about trans health and identities [55].

HCPs described uncertainty around trans bodies [37] which impeded their communication with patients and influenced their management decisions [38]. They observed patients be isolated on wards because of discrimination from HCPs and patients [37]. In addition, delays to care, inaccurate information [34], exclusion from screening services [32, 35], and avoidance of healthcare environments were reported [36]. Again, this is in line with patient perspectives [10] and similar findings from other marginalised groups [56]. Systemic and structural changes in healthcare institutions to become trans‐inclusive can reduce discrimination towards trans patients. Medical schools opposing conversion therapy for trans patients indicates that their institution is trans‐inclusive and contributes to a wider culture of equality for trans patients [57]. IT systems should be updated to allow name changes to minimise deadnaming and support appropriate pronouns. Secondary care providers can enforce policy against transphobia and consistent implementation of ward preference policies would demonstrate a trans‐inclusive approach. Without meaningful change, negative impacts on patient care will continue to have serious consequences for patient outcomes and add to health disparities between cis and trans patients.

### 5.2 Strengths

This review highlights the dearth of evidence on this topic and areas for future research and practice that may be valuable. Results were consistent across a range of healthcare settings, and the mostly qualitative findings offer in‐depth insight. Systematic search and extraction methods were used, enhancing methodological rigour. Finally, most of the articles for inclusion were of high quality as assessed by critical appraisal methods.

### 5.3 Limitations

Ideally, several reviewers would screen and extract data to limit bias however this is a student dissertation therefore this was not possible. Some of the studies in the review contained limited disaggregated data [35, 36, 40], and some excluded articles had an LGBTQ+ focus that did not disaggregate the data and could not be included, making this information inaccessible. When demographic information was available, several samples were predominantly comprised of straight, white women, which is not representative of the general population of UK HCPs [58, 59], which limits the transferability of the findings. Additionally, half of the article settings were focused on England only, limiting the transferability to a UK‐wide focus. Finally, the evidence is written from a Western lens, which does not account for cultural experiences and interpretations of gender identity outside of this paradigm [51].

## 6 Conclusion

This novel evidence review aimed to draw together and review existing evidence to better understand the themes around attitudes and knowledge of healthcare professionals towards transgender patients. The main themes are discriminatory attitudes (Theme 1), which were connected to knowledge gaps (Theme 2), which were linked with uncertainty (Theme 3), which ultimately can all negatively impact trans patients (Theme 4). Recommendations include embedding trans‐inclusive healthcare education in HCP curriculums and developing and implementing trans‐inclusive policy. It is hoped that this review contributes to moving towards a trans‐inclusive healthcare culture where trans patients feel welcome instead of stigmatised and can build trusting HCP–patient relationships with positive consequences for patient care.

## Author Contributions

**Amy Martin:** conceptualisation, investigation, methodology, formal analysis, project administration, writing – original draft, writing – review and editing. **Louise Preston:** conceptualisation, supervision, writing – review and editing.

## Funding

The authors have nothing to report.

## Conflicts of Interest

The authors declare no conflicts of interest.

## Supporting information


**Data S1:** Supporting Information.

## Data Availability

The data underlying this article are available from the corresponding author on reasonable request.
