# Supplementary material for: Identifying Themes in the Knowledge and Attitudes of UK Healthcare Professionals Towards Transgender and Nonbinary Patients: An Evidence Review
Source: Clin Teach. 2026 Apr 6;23(3):e70367. doi: 10.1111/tct.70367 (PMC13051252; doi:10.1111/tct.70367)
Supplement: Supplementary file 1 — Data S1: Supporting Information. [file TCT-23-e70367-s001.docx]

# Supplementary materials

## Supplementary material 1

### Search terms and search strategy

| Attitudes | “Attitude” OR “perception” OR “knowledge” OR “competency” OR “views” OR “awareness” OR “insights” OR “thoughts” OR “feelings” OR “opinions” OR “beliefs” OR “experiences” |
| --- | --- |
| Healthcare professionals | “Healthcare professional” OR “healthcare provider” OR “healthcare practitioner” OR “doctor” OR “nurse” OR “midwife” OR “trainee” OR “healthcare worker” OR “physician” OR “surgeon” OR “paediatrician” OR “psychiatrist” OR “clinician” OR “service provider” |
| Transgender | “transgender” OR “non-binary” OR “non binary” OR “LGBT” OR “transsexual” OR “transvestite” OR “genderqueer” OR “gender queer” OR “cross-dress” OR “cross dress” OR “gender diverse” OR “trans man” OR “gender reassignment” OR “gender-affirming” OR “gender affirming” OR “agender” OR “third gender” OR “sex change” OR “gender non-conforming” OR “gender minority” OR “trans men” OR “trans patient” OR “gender questioning” |

| **PsycINFO database** | | |
| --- | --- | --- |
|  | **Search** | **Results** |
| 1 | (transgender* or LGBT* or gender divers* or non-binary or genderqueer or gender affirm* or gender reassignment or trans patient* or trannsexual* or trans health*).ti. | 8050 |
| **2** | (doctor* or nurse* or midwi* or clinician* or health professional* or health care provider* or service provider* or practitioner* or surgeon* or paediatrician* or counsellor* or therapist* or psychiatrist*).ti. | 76258 |
| **3** | (attitude* or experience* or view* or perception* or belief* or insight* or opinion* or knowledge or awareness or competency).ti. | 406715 |
| **4** | #1 AND #2 AND #3 | 81 |

## Supplementary material 2

# References

1. Stonewall. Stonewall. 2019 [cited April 2024]. What does trans mean? Available from: <https://www.stonewall.org.uk/what-does-trans-mean>
2. Thelwall M, Devonport TJ, Makita M, Russell K, Ferguson L. Academic LGBTQ+ terminology 1900-2021: increasing variety, increasing inclusivity?. Journal of Homosexuality. 2023 Sep 19;70(11):2514-38.
3. ONS, 2023. Accessed August 2025. Gender identity, England and Wales, Census 2021. Available at: <https://www.ons.gov.uk/peoplepopulationandcommunity/culturalidentity/genderidentity/bulletins/genderidentityenglandandwales/census2021>.
4. Gwenffrewi G. The stoning of Stonewall during the new trans panic. [Internet]. Edinburgh; 2021 [cited April 2024]. Available from: https://www.ucu.org.uk/media/13245/The-stoning-of-Stonewall-during-the-new-trans-panic/pdf/The_stoning_of_Stonewall_Nov22.pdf
5. Wills M. JSTOR. 2020 [cited April 2024]. A History of Transphobia in the Medical Establishment. . Available from: https://daily.jstor.org/a-history-of-transphobia-in-the-medical-establishment/
6. Wright T, Nicholls EJ, Rodger AJ, Burns FM, Weatherburn P, Pebody R, et al. Accessing and utilising gender-affirming healthcare in England and Wales: trans and non-binary people’s accounts of navigating gender identity clinics. BMC Health Serv Res. 2021 Dec 28;21(1):609.
7. Willis P, Dobbs C, Evans E, Raithby M, Bishop J. Reluctant educators and self‐advocates: Older trans adults’ experiences of health‐care services and practitioners in seeking gender‐affirming services. Health Expectations. 2020 Oct 16;23(5):1231–40.
8. Ching BCF, Campbell A, Chase A, Schlief M, Hahn JS. Facilitation of sexual and gender identity disclosure and improved healthcare for LGBTQ+ patients: current processes, shortcomings, and recommendations for change. The British Student Doctor Journal. 2021 Apr 30;5(2):38.
9. Berner AM, Connolly DJ, Pinnell I, Wolton A, MacNaughton A, Challen C, et al. Attitudes of transgender men and non-binary people to cervical screening: a cross-sectional mixed-methods study in the UK. British Journal of General Practice. 2021 Aug;71(709):e614–25.
10. TransActual. TransActual. 2021 [cited April 2024]. Trans lives survey 2021: Enduring the UK’s hostile environment. . Available from: <https://www.transactual.org.uk/trans-lives-21>
11. Gov.UK. Gov.UK. 2019 [cited April 2024]. National LGBT survey: summary report. Available from: <https://www.gov.uk/government/publications/national-lgbt-survey-summary-report/national-lgbt-survey-summary-report>
12. Hennekam S, Dumazert J. Intersectional (in)visibility of transgender individuals with an ethnic minority background throughout a gender transition: Four longitudinal case studies. Gend Work Organ. 2023 Sep 6;30(5):1585–610.
13. Tollemache, N., Shrewsbury, D. and Llewellyn, C., 2021. Que (e) rying undergraduate medical curricula: a cross-sectional online survey of lesbian, gay, bisexual, transgender, and queer content inclusion in UK undergraduate medical education. *BMC Medical Education*, *21*(1), pp.1-12.
14. Barber, A.T., Flach, A.J. and Pattinson, E.M., 2022. A contemporary review of LGBTQ+ healthcare teaching in the UK medical curriculum. The British Student Doctor Journal, 6(1), pp.23-34.
15. Barber A, Flach A, Bonnington J, Pattinson EM. LGBTQ+ Healthcare Teaching in UK Medical Schools: An Investigation into Medical Students' Understanding and Preparedness for Practice. J Med Educ Curric Dev. 2023 Mar 27;10:23821205231164893. doi: 10.1177/23821205231164893. PMID: 37008793; PMCID: PMC10052488.
16. SGUL, 2023. *Transgender Healthcare: Caring for Trans Patients.* St George’s University of London. Accessed May 2023. Available online at: [https://www.futurelearn.com/courses/transgender-in-healthcare]
17. Flower, L., Cheung, A., Connal, S., Humphreys, A., Kamaruddin, K., Lennie, Y. and Edwardson, S., 2022. Management of transgender patients in critical care. *Journal of the Intensive Care Society*, p.17511437221145102.
18. GMC, (2021). *New guide for LGBT patients on what to expect from their doctor*. GMC. Accessed April 2023. Available online at: [https://www.gmc-uk.org/news/news-archive/new-guide-for-lgbt-patients-on-what-to-expect-from-their-doctor]
19. Khan F. Scientific American. 2016 [cited April 2024]. A history of transgender healthcare. Available from: <https://blogs.scientificamerican.com/guest-blog/a-history-of-transgender-health-care/>
20. Wills M. JSTOR. 2020 [cited April 2024]. A History of Transphobia in the Medical Establishment. Available from: <https://daily.jstor.org/a-history-of-transphobia-in-the-medical-establishment/>
21. Legislation.gov.uk. Legislation.gov.uk. 2010 [cited April 2024]. Equality Act 2010. Available from: <https://www.legislation.gov.uk/ukpga/2010/15/contents>
22. Human Rights Watch, 2019. *‘Don’t punish me for who I am’.* Human Rights Watch. Accessed online May 2023. Available at: [https://www.hrw.org/report/2019/09/03/dont-punish-me-who-i-am/systemic-discrimination-against-transgender-women-lebanon].
23. TransEDU. TransEDU. 2022 [cited April 2024]. Identity terms. Available from: <https://www.trans.ac.uk/ResourcesInformation/IdentityTerms/tabid/7237/Default.aspx>
24. Oregan Health and Science University. Oregan Health and Science University. . 2022 [cited April 2024]. Transgender health program: terms and tips. . Available from: <https://www.ohsu.edu/transgender-health/transgender-health-program-terms-and-tips>
25. Whittle S. The Guardian. 2010 [cited April 2024]. A brief history of transgender issues. . Available from: <https://www.theguardian.com/lifeandstyle/2010/jun/02/brief-history-transgender-issues>.
26. Lisik D, Ioannidou A, Milani G, Nvassi S, Ermis S, Spolidoro G, et al. JBI Data Extraction Form for Review for Systematic Reviews and Research Syntheses. Appendix 3: Data Extraction Form. BMJ Open. 2019 Jul;9(7).
27. Hong QN, Fàbregues S, Bartlett G, Boardman F, Cargo M, Dagenais P, et al. The Mixed Methods Appraisal Tool (MMAT) version 2018 for information professionals and researchers. Education for Information. 2018 Dec 18;34(4):285–91.
28. Tyndall J. Flinders University. 2010 [cited April 2024]. AACODS Checklist. . Available from: <http://dspace.flinders.edu.au/dspace/>
29. Braun V, Clarke V. Reflecting on reflexive thematic analysis. Qual Res Sport Exerc Health. 2019 Aug 8;11(4):589–97.
30. Byrne D. A worked example of Braun and Clarke’s approach to reflexive thematic analysis. Qual Quant. 2022 Jun 26;56(3):1391–412.
31. Canvin L. Narratives of mental health professionals supporting trans, gender diverse and gender questioning adults. . [Hertfordshire]: Univeristy of Hertfordshire; 2020.
32. Canvin L, Twist J, Solomons W. ”I don’t want to say the wrong thing”: mental health professionals’ narratives of feeling inadequately skilled when working with gender diverse adults. Psychol Sex. 2023 Apr 3;14(2):337–50.
33. Mikulak M. For whom is ignorance bliss? Ignorance, its functions and transformative potential in trans health. J Gend Stud. 2021 Oct 3;30(7):819–29.
34. Mikulak M, Ryan S, Ma R, Martin S, Stewart J, Davidson S, et al. Health professionals’ identified barriers to trans health care: a qualitative interview study. British Journal of General Practice. 2021 Dec;71(713):e941–7.
35. SIMPSON P, ALMACK K, WALTHERY P. ‘We treat them all the same’: the attitudes, knowledge and practices of staff concerning old/er lesbian, gay, bisexual and trans residents in care homes. Ageing Soc. 2018 May 29;38(5):869–99.
36. Bashir S, Fend M, Sarfraz MA. Medical attitudes towards transgender patients. Br J Hosp Med. 2023 Mar 2;84(3):1–6.
37. Somerville C. Unhealthy attitudes: the treatment of LGBT people within health and social care services. [Internet]. 2015 [cited April 2024]. Available from: <https://www.bl.uk/collection-items/unhealthy-attitudes-the-treatment-of-lgbt-people-within-health-and-social-care-services>
38. Lefkowitz ARF, Mannell J. Sexual health service providers’ perceptions of transgender youth in England. Health Soc Care Community. 2017 May;25(3):1237–46.
39. Kirlew MI, Lord H, Weber J. Exploring health and social care professionals’ initial perceptions of caring for trans patients. Nursing Standard. 2020 Sep 30;35(10):44–9.
40. Mollitt PC. Exploring cisgender therapists’ attitudes towards, and experience of, working with trans people in the United Kingdom. Couns Psychother Res. 2022 Dec 29;22(4):1013–29.
41. Brown M, McCann E, McLoughlin G, Martin CH, McCormick F. The views and experiences of midwifery academics regarding LGBTQ+ health education in pre-registration programmes in the United Kingdom and Ireland: Qualitative findings from a mixed-methods study. Nurse Educ Pract. 2023 Feb;67:103554.
42. Berner AM, Hughes DJ, Tharmalingam H, Baker T, Heyworth B, Banerjee S, et al. An evaluation of self-perceived knowledge, attitudes and behaviours of UK oncologists about LGBTQ+ patients with cancer. ESMO Open. 2020;5(6):e000906.
43. Braybrook D, Bristowe K, Timmins L, Roach A, Day E, Clift P, et al. Communication about sexual orientation and gender between clinicians, LGBT+ people facing serious illness and their significant others: a qualitative interview study of experiences, preferences and recommendations. BMJ Qual Saf. 2023 Feb;32(2):109–20.
44. Mollitt P. BACP. 2022 [cited April 2024]. The big issue: Fear, anxiety and the toxic trans debate. . Available from: <https://www.bacp.co.uk/bacp-journals/therapy-today/2022/june-2022/the-big-issue/>
45. Owen-Pugh V, Baines L. Exploring the clinical experiences of novice counsellors working with LGBT clients: Implications for training. Couns Psychother Res. 2014 Mar 11;14(1):19–28.
46. NHS England. NHS England. 2019 [cited April 2024]. Delivering same sex accommodation. . Available from: <https://www.england.nhs.uk/statistics/wp-content/uploads/sites/2/2021/05/NEW-Delivering_same_sex_accommodation_sep2019.pdf>
47. BMA. British Medical Association. 2020 [cited April 2024]. Motion 4: Healthcare and rights of transgender and nonbinary individuals. . Available from: <https://www.bma.org.uk/media/3264/motion-4-healthcare-and-rights-of-transgender-and-nonbinary-individuals-briefing-arm-2020.pdf>
48. EHRC, 2025. An interim update on the practical implications of the UK supreme court judgement. Accessed August 2025. Available at: [https://www.equalityhumanrights.com/media-centre/interim-update-practical-implications-uk-supreme-court-judgment]
49. Nursing and Midwifery Council. Nursing and Midwifery Council. . 2023 [cited April 2024]. The code. Available from: <https://www.nmc.org.uk/globalassets/sitedocuments/nmc-publications/nmc-code.pdf>
50. St Catherine University. St Catherine University. 2022 [cited April 2024]. Health Equity vs. Health Equality: What’s the Difference? . Available from: <https://www.stkate.edu/academics/healthcare-degrees/health-equity-vs-health-equality>
51. Heng A, Heal C, Banks J, Preston R. Transgender peoples’ experiences and perspectives about general healthcare: A systematic review. International Journal of Transgenderism [Internet]. 2018 Oct 2 [cited 2024 Aug 5];19(4):359–78. Available from: <https://www.tandfonline.com/doi/full/10.1080/15532739.2018.1502711>
52. Holland D, White LCJ, Pantelic M, Llewellyn C. The experiences of transgender and nonbinary adults in primary care: A systematic review. European Journal of General Practice [Internet]. 2024 Dec 31 [cited 2024 Aug 5];30(1). Available from: <https://www.tandfonline.com/doi/full/10.1080/13814788.2023.2296571>
53. Thorne N, Yip AKT, Bouman WP, Marshall E, Arcelus J. The terminology of identities between, outside and beyond the gender binary – A systematic review. International Journal of Transgenderism. 2019 Jul 3;20(2–3):138–54.
54. Wray J, Guseppe A. The importance of Continuing professional development (CPD) for nurses. . BMJ Open [Internet]. 2021 [cited 2023 Dec 21]; Available from: <https://blogs.bmj.com/ebn/2021/11/14/the-importance-of-continuing-professional-development-cpd-for-nurses/>]
55. Safer JD, Pearce EN. A Simple Curriculum Content Change Increased Medical Student Comfort with Transgender Medicine. Endocrine Practice. 2013 Jul;19(4):633–7.
56. Salkind, Gishen, Drage, Kavanagh, Potts. LGBT+ Health Teaching within the Undergraduate Medical Curriculum. Int J Environ Res Public Health. 2019 Jun 28;16(13):2305.
57. Braun HM, Garcia-Grossman IR, Quiñones-Rivera A, Deutsch MB. Outcome and Impact Evaluation of a Transgender Health Course for Health Profession Students. LGBT Health. 2017 Feb;4(1):55–61.
58. Hall, W. J., Chapman, M. v, Lee, K. M., Merino, Y. M., Thomas, T. W., Payne, B. K., Eng, E., Day, S. H., & Coyne-Beasley, T. (2015). Implicit Racial/Ethnic Bias Among Health Care Professionals and Its Influence on Health Care Outcomes: A Systematic Review. *American Journal of Public Health*, *105*(12), e60-76. <https://doi.org/10.2105/AJPH.2015.302903>
59. GLADD. GLADD. 2023 [cited April 2024]. So-called “conversion therapy”. Medical schools charter. Available from: <https://gladd.co.uk/activism-conversion-therapy-charter/>
60. NHS England. NHS England. 2023 [cited April 2024]. New figures show NHS workforce most diverse it has ever been. Available from: https://www.england.nhs.uk/2023/02/new-figures-show-nhs-workforce-most-diverse-it-has-ever-been/
61. NHS Employers. NHS Employers. 2019 [cited April 2024]. Gender in the NHS infographic. . Available from: https://www.nhsemployers.org/articles/gender-nhs-infographic
